# Detection of Neutralizing Antibodies to Tembusu Virus: Implications for Infection and Immunity

**DOI:** 10.3389/fvets.2019.00442

**Published:** 2019-12-10

**Authors:** Junfeng Lv, Lixin Yang, Shenghua Qu, Runze Meng, Qingxiangzi Li, Huicong Liu, Xiaoyan Wang, Dabing Zhang

**Affiliations:** Key Laboratory of Animal Epidemiology and Zoonosis of Ministry of Agriculture, College of Veterinary Medicine, China Agricultural University, Beijing, China

**Keywords:** duck, Tembusu virus, TMUV infection, TMUV attenuated vaccine, humoral immune response, neutralizing antibody, plaque reduction neutralization test

## Abstract

Neutralizing antibodies are the key mediators of protective immune response to flaviviruses after both infection and vaccination. Plaque reduction neutralization test (PRNT) is considered the “gold standard” for measurement of the immunity. To date, little is known regarding neutralizing antibody response to Tembusu virus (TMUV), a novel flavivirus emerging in ducks in 2010. Here, we developed a PRNT for detection of TMUV neutralizing antibodies. Following optimization and validation, the PRNT was applied to test serum samples from different flocks of ducks. Using sera prepared in experimental conditions, the levels of 50% end point titer (neutralizing dose, ND_50_) generated from positive sera (5,012–79,433) were significantly higher than those from mock-infected sera (10 to 126), indicating that the test can be used in the detection of TMUV-specific neutralizing antibodies. Dose-dependent efficacy test of a cell-derived 180th passage of a plaque-purified virus of the PS TMUV isolate (PS180) in combined with immunization-challenge experiments revealed that ND_50_ titer of ~1,258 is the minimum capable of providing adequate protection against challenge with virulent TMUV. In the investigation of serum samples collected from three flocks infected by TMUV and four flocks vaccinated with a licensed attenuated vaccine (the 120th passage virus), ND_50_ titers peaked at 1 week after both disease onset (7,943–125,893) and vaccination (3,612–79,432), and high levels of ND_50_ titer were detected in sera collected at 15 weeks after disease onset (5,012–63,095) and 17 weeks after vaccination (3,981–25,119). Together these findings demonstrated that spontaneous and experimental infections by TMUV and vaccination with the licensed TMUV attenuated vaccine elicit high, long-lasting neutralizing antibodies. The highest ND_50_ titer of neutralizing antibodies elicited by PS180 was determined to be 3,162, suggesting that attenuation of TMUV by more passages has a dramatic impact on the neutralizing antibody response of the virus.

## Introduction

Humoral immune response plays a significant role in protection of the host from flavivirus infections (1). Generally, neutralizing antibodies are thought to be the key mediators of protection against flaviviruses following both infection and vaccination (2, 3). Under some circumstances, however, antibodies may enhance flavivirus infection, a phenomenon called antibody-dependent enhancement (ADE) of infection (4, 5). In the case of dengue virus (DENV), the ADE phenomenon most frequently occurs in secondary infections with different DENV serotypes and in children with maternal antibodies declined to sub-neutralizing concentrations (6, 7). In fact, any antibody that neutralizes at sufficiently high concentrations can enhance flavivirus infectivity at sub-neutralizing concentrations (3). Therefore, high levels of neutralizing antibody are crucial to exerting a protective effect, especially in the context of vaccine-mediated humoral immunity.

For assessing the host's specific immune response to flaviviruses, many useful serological tests have been developed, such as hemagglutination inhibition, complement fixation, enzyme-linked immunosorbent assay (ELISA), and plaque reduction neutralization test (PRNT) (8). However, flavivirus serological tests may present a classical challenge in differential diagnosis owing to strong cross-reactivity between antibodies and heterologous viral antigens (9, 10). PRNT measures neutralizing antibodies, and is the most virus-specific serological test. Thus, this test is considered the “gold standard” for the differentiation of flavivirus infections and the measurement of immunity to flaviviruses although it is time-consuming to perform (2, 8, 11–18).

Tembusu virus (TMUV) is currently classified within the genus *Flavivirus* of the family *Flaviviridae* (https://talk.ictvonline.org/taxonomy). Based on the mode of transmission and serological cross-reactivity, TMUV is also classified within the Ntaya group of the mosquito-borne flavivirus group, along with Bagaza virus (BAGV), Ntaya virus (NTAV), and Zika virus (ZIKV) (8). TMUV-related disease in ducks emerged in 2010, which affects mainly ducks during egg-laying periods. The disease is characterized by sudden onset, rapid spread, severe drops in egg production, and degenerate ovaries with hemorrhagic lesions (19–22). In affected flocks, the egg production rate may reduce to 10% or less within ~1 week after disease onset (23). To control the disease, several vaccine candidates have been developed, including live-attenuated (24), inactivated (25, 26), and subunit-based (27–31) vaccine candidates. Live-attenuated and inactivated vaccines have been licensed to use in ducks in China (32, 33).

It has been shown previously that humoral immune response to TMUV can be developed in ducks following vaccination with various vaccine candidates as described above. Most data regarding to antibody response were generated by using ELISA-based assays, such as indirect ELISA (34), competition ELISA (35), and blocking ELISA (36). In the study by Chen et al. (27), a neutralization test was applied to detect serum antibodies of ducks vaccinated with a vectored duck enteritis virus expressing the TMUV envelope. That investigation showed that the vaccine candidate elicits neutralizing antibodies, with titers of 1:2^8^ at 7 days after immunization and 1:2^4^ at 15 weeks after immunization. A serological investigation performed on 60 serum samples collected from six farms by using a blocking ELISA revealed a high prevalence of 56.7% (36). To date, data relating to TMUV neutralizing antibody response elicited by infection and vaccine is still limited.

In this study, we describe the development of a PRNT for the detection of TMUV neutralizing antibodies. We also describe the application of the test to field serum samples from different flocks of diseased and immunized ducks.

## Materials and Methods

### Cells

BHK-21 cells were maintained in growth medium consisting of Dulbecco's Modified Eagle Medium (DMEM; Gibco, NY, USA) supplemented with 10% fetal calf serum (FCS; Gibco, NY, USA), 100 U/ml penicillin, and 100 μg/ml streptomycin.

### Viruses

The PS and Y isolates were originally recovered in China from outbreaks of TMUV-related disease in a flock of egg-laying ducks in 2011 and in a flock of 74-day-old ducks, respectively. The fourth passage (strain PS4) in BHK-21 cells of the PS isolate was applied to produce the working virus in PRNT. The fourth passage (strain Y4) in BHK-21 cells of the Y isolate was applied to produce TMUV antibody-positive sera. The 180th passage (strain PS180) of a plaque-purified virus of the PS isolate was used in immunization-challenge experiments. The PS180 virus was prepared in our laboratory, which underwent five passages in 9-days-old specific pathogen free chicken embryos, three passages in BHK-21 cells, three-rounds of plaque purification, and 180 passages in BHK-21 cells (37, 38).

### Virus Propagation

BHK-21 cells were prepared in T25 flasks, which were seeded at 8 × 10^6^ cells/flask. When confluent, the cells were washed three times with phosphate buffered solution (PBS), and inoculated with PS4 at a multiplicity of infection (MOI) of 0.01 plaque forming unit (PFU)/cell. Following 1-h adsorption at 37°C, the inoculum was removed, and 5 ml of maintenance medium consisting of DMEM supplemented with 2% FCS, 100 U/ml penicillin and 100 μg/ml streptomycin was added to each flask. The cells were incubated at 37°C in a 5% CO_2_ atmosphere. At about 60 h post inoculation (pi), when a remarkable cytopathic effect (CPE) was developed, the cell culture was subjected to a cycle of freeze and thawing, followed by centrifugation at 10,000 × g for 1 min. Cell-free supernatant was harvested for additional three passages, generating the PS5, PS6, PS7, and PS8 strains. The viruses were stored at −80°C until use.

### Serum Samples

TMUV antibody-negative and positive sera were prepared in Pekin ducklings (*Anas platyrhynchos domestica*) and used for the development of PRNT. The newly hatched ducklings were obtained from a local duck farm, where TMUV infection had never been observed and their parents had never been immunized with TMUV vaccine. Antisera (*n* = 10) against Y4 were taken from 14-days-old survived ducklings that were inoculated intramuscularly (i.m.) with strain Y4 at 7 days of age at the dose of 10^4^ PFU per duckling. The TMUV antibody-negative sera were collected from 1-day-old ducklings (*n* = 10), and also prepared from 14-days-old ducklings (*n* = 10) which were inoculated i.m. with 0.2 ml of DMEM at 7 days of age.

Three groups of serum samples collected from ducks showing drops in egg production were used for investigation of the levels of neutralizing antibody induced by TMUV infection. The first group consisted of 20 samples collected in Inner Mongolia autonomous region from 29-weeks-old Pekin ducks in which the egg drop disease had been observed at 28 weeks of age. The egg laying rate decreased by 20–30%, lower than those caused by TMUV infection (23). Using previously reported TMUV-specific reverse transcription (RT)-PCR assays (19, 20), no positives were found in theca folliculi of diseased ducks. The second group consisted of 50 samples collected at 1 week after disease onset, including 20 samples taken in Hubei province from 28-weeks-old Shaoxing duck (*Anas platyrhynchos domestica*) and 30 samples taken in Shandong province from 31-weeks-old Cherry Valley Pekin ducks (a strain of Pekin duck; *Anas platyrhynchos domestica*). The presence of TMUV infection in the flocks were confirmed based on clinical features and detection of TMUV in theca folliculus samples by using the RT-PCR assays (19, 20). The third group comprised 75 samples collected in Liaoning province from a flock of Jinding ducks (*Anas platyrhynchos domestica*). In the flock, a severe drop in egg production was observed at 25-weeks-old. TMUV was detected in theca folliculus samples. The serum samples were taken at 1, 3, 7, 11, and 15 weeks after disease onset. At each sampling time point, 15 samples were collected.

Two groups of serum samples collected from immunized ducks were used for investigation of the levels of neutralizing antibody induced by immunization with a licensed TMUV live-attenuated vaccine, the 120th passage of the WF TMUV isolate (32). The first group consisted of 110 samples collected at 1 week after immunization, including 30 samples collected in Anhui province from 19-weeks-old Cherry Valley Pekin ducks, 30 samples collected in Shandong province from 21-weeks-old Cherry Valley Pekin ducks, and 50 samples collected in Liaoning province from 21-weeks-old Jinding ducks. The second group comprised 70 serum samples, which were collected in Shandong province from a flock of Cherry Valley Pekin ducks. The ducks were vaccinated at 20 weeks of age. The samples were taken at 1 week before immunization, and at 1, 3, 5, 9, 13, and 17 weeks after immunization. At each sampling time point, 10 samples were collected. No clinical signs were observed during the course of sampling.

### Plaque Assay

The virus was prepared in serial 10-fold dilutions (10^−1^–10^−6^) with DMEM. BHK-21 cells were cultured on 24-well plates (Corning, NY, USA), which were seeded at 4 × 10^5^ cells/well. When confluent, the cells were washed three times with PBS, and inoculated with 0.1 ml of diluted virus, three cultures per dilution. Following 1 h adsorption at 37°C, the inoculum was removed and the cells were washed three times with PBS. The plates received 0.5 ml of overlay medium consisting of DMEM containing 2% FCS, 100 U/ml penicillin, 100 μg/ml streptomycin and 1% low melting-point agarose (Macgene, Beijing, China), and placed at 4°C for 15 min for agarose coagulation. After 3 days further incubation at 37°C in a 5% CO_2_, the cells were fixed with 0.5 ml of 4% paraformaldehyde at room temperature for 90 min. The paraformaldehyde and agarose were removed and the cells were stained with 0.5 ml of 0.2% (w/v) crystal violet. Fifteen minutes later, crystal violet was removed and plaques were read. Virus titer was calculated as described previously (39).

### Growth Kinetics

Confluent monolayers of BHK-21 cells cultured in T25 flasks were inoculated with TMUV at a MOI of 0.01 PFU/cell as described above. For assessing virus growth, medium was sampled every 12 h between 12 and 84 hpi. At each sampling point, 0.2 ml sample was collected. Virus titers were determined using plaque assay as described above.

### Development of PRNT

The PS7 strain was used as a working virus to develop PRNT for the detection of TMUV-specific neutralizing antibodies in sera of ducks. Ten antisera against the Y4 isolate and 10 sera from 1-day-old ducklings were tested in a preliminary protocol. Six antisera with 50% end point titers (neutralizing dose, ND_50_) ranging from 10,000 to 125,893 and four sera with ND_50_ titers ranging from 10 to 125 were selected for optimization of the PRNT. To determine the optimal dilution of working virus, four different dilutions (1:5 × 10^3^, 1:7 × 10^3^, 1:10^4^, and 1:2 × 10^4^) of the PS7 virus were tested against the 10 sera. Subsequently, varying incubation times around the following steps of PRNT were tested: (i) virus-serum neutralization (30, 45, 60, 75, and 90 min); (ii) adsorption of virus-serum inoculum to the cell surface (30, 40, 50, and 60 min); (iii) incubation after the plates received overlay medium (60, 66, 72, 78, and 84 h). For each condition, each serum sample was tested three times, generating three ND_50_ titers (the observed results) and one median titer. The optimal condition was confirmed if all serum samples tested had the observed results within one 3-fold difference of the median titer. For determination of the optimal dilution of working virus and the optimal incubation time after the plates received overly medium, the conditions to get readily discernable plaques and minimize plaque overlap were considered to be acceptable.

### PRNT Protocol

Serum was inactivated at 56°C for 30 min and diluted in a 10-fold step to 10^−5^ with DMEM. Each dilution of the sera was mixed with an equal volume of diluted virus, and incubated at 37°C for 1 h. BHK-21 cell cultures were prepared in 24-well plates as described above. When confluent, the cells were washed and inoculated with 0.2 ml of the virus-serum mixtures, and plaque assay was conducted as described above. Controls were included in the experiment, including three virus control groups, in which 0.1 ml of virus was used to infect BHK-21 cells, and an uninfected BHK-21 cell control group, in which 0.1 ml of DMEM was used as inoculum. The PRNT titer was calculated using the Kärber method and expressed as ND_50_. The formula is: log_10_ ND_50_ = m – Δ (∑p – 0.5), where m indicates log_10_ of the highest dilution, Δ indicates log_10_ of dilution coefficient, and ∑p indicates the sum of number of plaques produced by virus-serum inoculum/average number of plaques produced by virus controls (40, 41).

### Validation of PRNT

The optimized PRNT assay was validated as described previously (42). The precision of the assay was assessed by using 20 TMUV antibody-negative sera and 10 antisera against Y4. The intra-assay precision was determined by testing a single sample in three independent assays by the same operator, and the inter-assay precision was determined by testing a single sample in three independent assays by three operators. The sensitivity of the test was evaluated using 10 antisera against Y4. The serum samples were individually diluted in DMEM at a 10-fold step to 10^−3^. Each of undiluted and diluted samples was tested three times by using the optimized PRNT.

### Detection of TMUV Neutralizing Antibodies in Serum Samples of Ducks

The PRNT was applied to test serum samples of ducks collected in the field. To investigate the neutralizing antibody response induced by PS180, vaccination experiments were conducted. Sixty 1-day-old Pekin ducklings, which were derived from non-immune breeders that had no antibody to TMUV, were randomly divided into six groups (10 birds/group). Four groups were vaccinated i.m. with PS180 at doses of 10^4^ (designated PS180-10^4^), 10^3^ (PS180-10^3^), 10^2^ (PS180-10^2^), and 10 (PS180-10) PFU per ducklings, respectively. Two groups (mock and control) were inoculated with 0.2 ml of DMEM. The birds were separately reared in different isolators. Serum samples were taken from all ducklings at 7 days p.i. and tested for neutralizing antibodies by using PRNT.

### Vaccination and Challenge Experiments

To assess the immunogenicity of TMUV PS180, the vaccinated birds were challenged i.m. with TMUV Y4 (10^5^ PFU per duckling) at 7 days p.i. after serum collection. The groups were designated PS180-10^4^/Y4, PS180-10^3^/Y4, PS180-10^2^/Y4, and PS180-10/Y4, respectively. Of the two unvaccinated groups, one (mock/Y4) was challenged as described above, and another (control) was inoculated i.m. with 0.2 ml of DMEM. Ducklings were monitored daily for 7 days. Living ducks were weighed at the end of the experiment.

### Statistical Analysis

The data derived from growth kinetic analysis, PRNT, and immunization-challenge experiments were calculated at mean ± standard deviation (SD). Difference between groups were compared using the analysis of variance method implemented in the GraphPad Prism Software (Version 5.0; GraphPad Software Inc., San Diego, CA, USA). A *P* < 0.05 value was considered statistically significant.

## Results

### The Seventh Passage of TMUV PS in BHK-21 Cells Was Selected as Working Virus

It has been shown previously that the fifth to tenth passages of cell-derived virus are generally used as working virus in the PRNT assay (13). To produce the working virus for TMUV PRNT, the PS4 strain was passaged for four times in BHK-21 cells and the growth properties of the resulting four strains (PS5–PS8) were compared.

**Figure 1:**
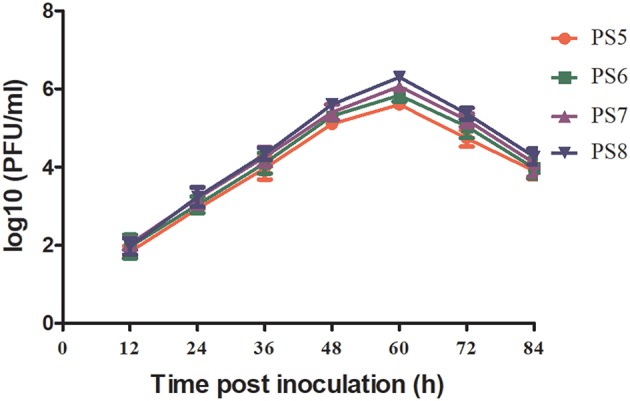
TMUV growth kinetics in BHK-21 cells. Virus titers in plaque-forming units (PFU)/ml were measured between 12 and 84 hpi with a 12 h interval.

**Figure 2:**
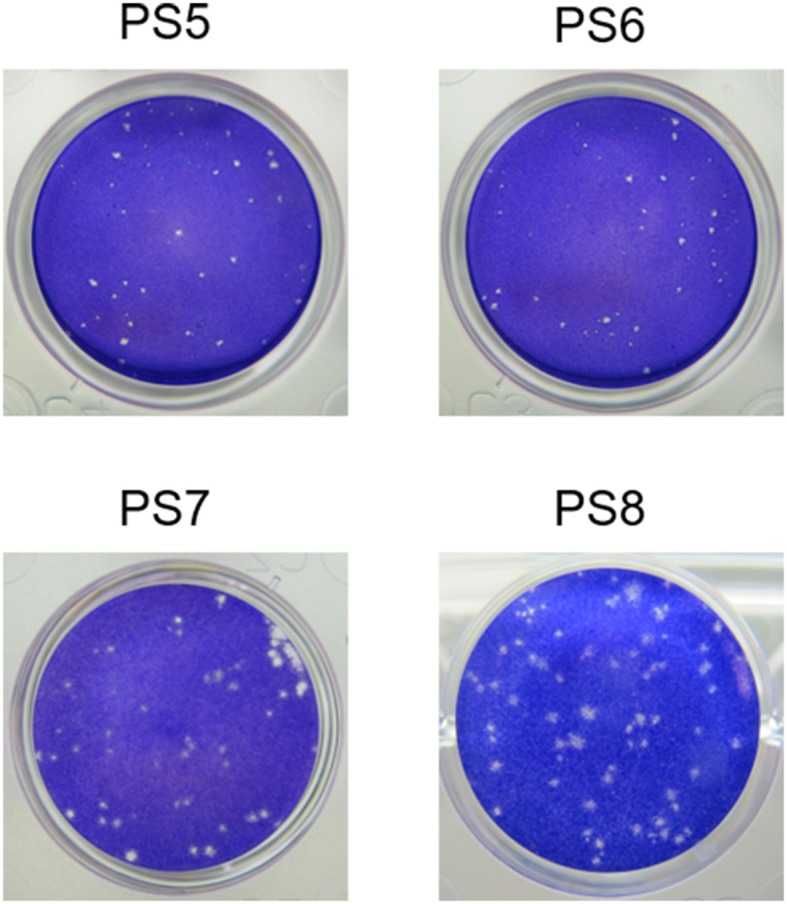
TMUV plaques in BHK-21 cells at 72 h post inoculation.

All the four viruses grew well in BHK-21 cells and exhibited similar growth kinetics (Figure 1). The titers of the four viruses showed increase since 12 hpi, and peaked at 60 hpi. Since then, the titers of the viruses tended downwards. During the observation period, the titers of PS7 and PS8 were slightly higher than those of PS5 and PS6, whereas no significant differences were observed between the viruses (*P* > 0.05). All the viruses produced clear plaques in BHK-21 cells. Differences were detected in plaque sizes, with 1–2 mm in diameter in the PS7- an PS8-infected cells and <1 mm in diameter in the PS5- and PS6-infected cells (Figure 2).

Based on above observations, the PS7 isolate was used as working virus in TMUV PRNT. Thus, the working virus was produced by infecting BHK-21 cells with the PS6 strain. The PS7 virus was harvested at 60 hpi, dispensed into small aliquots, and stored at −80°C. The titer was determined to be 4.2 × 10^6^ PFU/ ml.

### The TMUV Antibody-Positive Sera Can Be Clearly Distinguished From the TMUV Antibody-Negative Sera by the PRNT

The 1:5 × 10^3^ dilution of the working virus produced ~80 plaques/well, which had an impact on the accuracy of counting. The 1:7 × 10^3^, 1:10^4^, and 1:2 × 10^4^ dilutions of the working virus produced ~60, 40, and 20 plaques/well, respectively, all of which assured a good precision of counting. In PRNT using 1:7 × 10^3^ and 1:10^4^ dilutions of the working virus, all serum samples tested showed the three observed values within one 3-fold difference of the median ND_50_ titer (Supplementary Table S1). 1:10^4^ was selected as the optimal dilution of the working virus. On this basis, the 45, 60, and 75 min neutralization times and the 40, 50, and 60 min adsorption times were all shown to assure the accuracy of PRNT (Supplementary Tables S2, S3). Sixty minutes, which was generally employed in the routine neutralization test, was regarded as the optimal incubation time for both neutralization and adsorption. After the plates received overlay medium, the 72, 78, and 84 h incubation times were all considered to be acceptable as plaques with 1–2 mm in diameter formed at these time points were easily recognized and counted (Supplementary Figure S1). Seventy-two hours was selected as the optimal incubation time.

As shown in Tables 1, 2, there were only three and two samples exhibited results that were outside ± 3-fold of the median ND_50_ titer of the three results in the intra- and inter-assay precision analyses, respectively. In the sensitivity analysis, all the samples were shown to have an observed and expected ND_50_ titer within 3-fold of each other. For all serum samples tested, antibodies were detectable at a 1:100 dilution, but not at a 1:1,000 dilution (Table 3). In the light of criteria previously defined for DENV (42), the precision of intra- and inter-assays and the sensitivity of the TMUV PRNT were considered acceptable.

**Table 1:** Determination of intra-assay precision for TMUV PRNT.

**Sample no**.	**ND**_****50****_ **for antibody-negative sera^†^**			**Sample no**.	**ND**_****50****_ **for antibody-negative sera^‡^**			**Sample no**.	**ND**_****50****_ **for anti-Y4 sera**		
	**Test 1**	**Test 2**	**Test 3**		**Test 1**	**Test 2**	**Test 3**		**Test 1**	**Test 2**	**Test 3**
1	10	16	10	1	13	10	10	1	12,589	15,848	7,943
2	13	20	20	2	25	16	20	2	6,309	10,000	7,943
3	50	63	40	3	25	13	32	3	50,118	31,622	39,811
4	13	16	12	4	32	40	40	4	7,943	10,000	12,589
5	63	79	50	5	126	100	63	5	31,622^*^	10,000	10,000
6	32	25	40	6	100	79	79	6	5,012	6,309	10,000
7	126	79	63	7	100	79	25^*^	7	15,849	19,953	15,849
8	100	126	79	8	63	79	50	8	25,119	25,119	39,811
9	32	50	63	9	100	100	126	9	63,096	79,433	19,953^*^
10	13	16	10	10	40	32	32	10	10,000	12,589	15,849

† *Sera collected from 1-day-old ducklings*.

‡ *Sera prepared from 14-days-old ducklings that had been inoculated with DMEM at 7 days of age*.

* *Outside ± 3-fold of the median titer of three results obtained in triplicates*.

**Table 2:** Determination of inter-assay precision for TMUV PRNT.

**Sample no**.	**ND**_****50****_ **for antibody-negative sera^†^**			**Sample no**.	**ND**_****50****_ **for antibody-negative sera^‡^**			**Sample no**.	**ND**_****50****_ **for anti-Y4 sera**		
	**Test 1**	**Test 2**	**Test 3**		**Test 1**	**Test 2**	**Test 3**		**Test 1**	**Test 2**	**Test 3**
1	10	20	25	1	13	20	13	1	12,589	10,000	10,000
2	13	16	20	2	25	20	20	2	6,309	7,943	31,623^*^
3	50	64	50	3	20	10	20	3	50,118	39,811	63,096
4	13	25	16	4	32	25	32	4	7,943	10,000	15,849
5	63	79	63	5	100	79	50	5	31,622	39,811	15,849
6	32	40	50	6	126	32^*^	63	6	5,012	12,589	7,943
7	126	100	79	7	63	79	79	7	15,849	19,953	19,953
8	100	50	63	8	100	50	63	8	25,119	31,622	19,953
9	32	40	63	9	79	50	50	9	63,096	50,119	79,433
10	13	20	10	10	40	50	25	10	10,000	19,953	15,849

† *Sera collected from 1-day-old ducklings*.

‡ Sera prepared from 14-days-old ducklings that had been inoculated with DMEM at 7 days of age.

* *Outside ± 3-fold of median titer of three results obtained in triplicates*.

**Table 3:** Evaluation of the sensitivity for TMUV PRNT.

**Sample no**.	**ND**_****50****_ **titer of neutralizing antibody in serum sample with dilution indicated**						
	**Undiluted**	**1:10**		**1:100**		**1:1,000**	
	**Median^**^†^**^**	**Median**	**Expected^**^‡^**^**	**Median**	**Expected**	**Median**	**Expected**
1	39,811	5,012	3,981	794	398	20	40
2	7,943	1,000	794	50	79	16	8
3	15,849	3,162	1,585	79	159	13	16
4	15,849	1,995	1,585	100	159	50	16
5	50,119	6,310	5,012	316	501	63	50
6	15,848	1,000	1,585	200	159	20	16
7	12,589	1,000	1,259	79	126	32	13
8	31,622	5,012	3,162	200	316	63	32
9	12,589	2,511	1,259	126	126	25	13
10	79,433	7,943	7,943	501	794	125	80

† *Median titer, mean value of three titers obtained in triplicates from each of diluted serum samples*.

‡ *Expected titer, median titer of the undiluted sample divided by the dilution factor*.

The 10 antisera against Y4 produced high levels of ND_50_ titer, ranging from 5,012 to 79,433 (Tables 1, 2). In sharp contrast to the antisera, the 10 sera collected from 1-day-old ducklings and the 10 sera prepared from 14-days-old ducklings that had been inoculated with DMEM produced significantly lower ND_50_ values, ranging from 10 to 126 (Tables 1, 2). These data indicated that the TMUV antibody-positive sera can be distinguished clearly from the TMUV antibody-negative sera by the optimized PRNT.

### The Cell-Derived PS180 Virus Exhibits Satisfied Immunogenicity

To assess the immunogenicity of the cell-derived 180th passage of PS virus (PS180), groups of one-day-old Pekin ducklings were vaccinated with PS180 at different doses and challenged with virulent TMUV (Y4) seven days later. No clinical signs were observed in control and ducks that had been vaccinated with 10^4^ and 10^3^ PFU of PS180. In contrast, ducks in other groups developed signs of illness at 3 days after challenge. Mild signs, such as mild depression and a slight decrease in feed intake, were observed in ducklings vaccinated with 10^2^ PFU of PS180. More serious signs, including dramatic decrease in feed intake, paralysis, and encephalitis, were seen in ducklings vaccinated with 10 PFU of PS180 and unvaccinated ducklings (Table 4).

**Table 4:** Correlation between levels of neutralizing antibody and protective efficacies conferred by TMUV PS180.

**Group^†^**	**ND_**50**_ titer**	**Mortality (%)**	**Mean body weight (*g*)^§^**
PS180-10^4^/Y4	1,584–3,162	0 (0/10)^‡^	572 ± 20
PS180-10^3^/Y4	1,258–2,511	0 (0/10)	560 ± 30
PS180-10^2^/Y4	631–1,548	10 (1/10)	450 ± 26^*^
PS180-10/Y4	251–398	40 (4/10)	414 ± 50^*^
Mock/Y4	25–158	50 (5/10)	400 ± 39^*^
Control	32–126	0 (0/10)	580 ± 18

† *Pekin ducks were inoculated with 10^4^ to 10 PFU of PS180 and DMEM at 1 day of age. Ducks in five groups were challenged with 10^5^ PFU of TMUV Y4 at 7 days of age. Serum was sampled from 7-days-old ducks before challenge*.

‡ *No. of death/no. of ducks challenged*.

§ *The ducks were weighed at 7 days after challenge*.

* *Significant difference compared with control (P < 0.05)*.

One duckling vaccinated with 10^2^ PFU of PS180 died within 6–7 days post challenge (p.c.); four ducklings vaccinated with 10 PFU of PS180 died within 6–7 days p.c.; five unvaccinated ducklings died within 5–7 days p.c. In contrast, no deaths occurred in control and ducklings that had been vaccinated with 10^4^ or 10^3^ PFU of PS180. These data indicated that one-day-old ducklings immunized i.m. with PS180 at higher doses (10^4^ and 10^3^ PFU) withstood an i.m. challenge of 10^5^ PFU of virulent TMUV (Y) 7 days later, whereas 10^2^ and 10 PFU of PS180 did not offered adequate protection (Table 4).

The body weights of living ducks in vaccinated and unvaccinated groups at 7 days after challenge were measured to investigate further the immunogenicity of PS180 in terms of weight gain. Challenge had no significant impact on weight gain in ducks vaccinated with 10^4^ and 10^3^ PFU of PS180 when compared with control (*P* > 0.05). In contrast, mean body weights of ducks immunized with 10^2^ and 10 PFU of PS180 and unvaccinated ducks were all significantly lower than that of control (*P* < 0.05) (Table 4). Taken together, it is concluded that PS180 exhibits satisfied immunogenicity.

### Neutralizing Antibodies With a ND_50_ Titer of Higher Than 1,258 Provides Adequate Protection Against Infection With Virulent TMUV

To define the minimal value of TMUV neutralizing antibodies protecting ducks against TMUV infections, serum samples were collected before challenge in above trials for vaccination-challenge experiments and tested for TMUV neutralizing antibodies by using PRNT (Table 4). Higher ND_50_ values, ranging from 1,584 to 3,162 and 1,258 to 2,511, were derived from sera of ducklings vaccinated with 10^4^ and 10^3^ PFU/bird PS180, respectively. Relatively low ND_50_ values, ranging from 631 to 1,548 and 251 to 398, were detected in sera of ducks after immunization with 10^2^ and 10 PFU of PS180, respectively. The levels of ND_50_ titer detected in sera taken from unvaccinated ducks were very low, ranging from 25 to 158, which were considered TMUV antibody-negative according to the criterion defined above. These data, in conjunction with results derived in the immunization-challenge experiments, suggested that ND_50_ titer of ~1,258 can be considered the minimum capable of providing adequate protection against challenge with virulent TMUV.

### TMUV Natural Infection Elicits High-Level, Long-Term Neutralizing Antibody Response in Ducks

**Figure 3:**
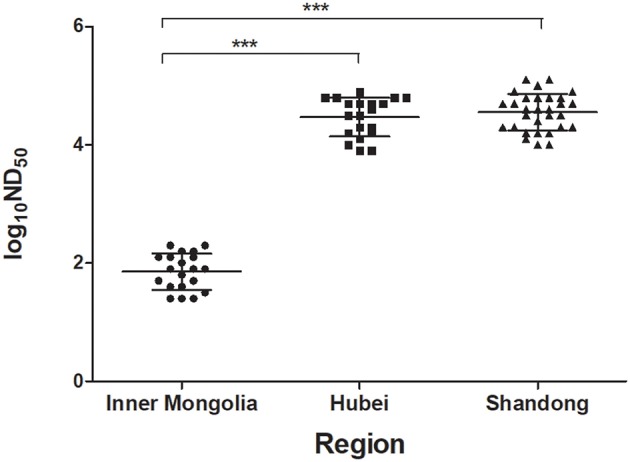
Testing of sera of ducks showing drops in egg production by using PRNT. The serum samples were collected at 1 week after disease onset from three flocks of ducks in different regions. Based on clinical features and molecular detection, TMUV infections had been confirmed to be responsible for the egg drop disease occurred in ducks in Hubei and Shandong provinces, but not for the disease occurred in ducks in Inner Mongolia autonomous region. ***, significant difference in antibody titer between Hubei and Inner Mongolia and between Shandong and Inner Mongolia (*P* < 0.001).

To investigate the humoral immune response induced by TMUV natural infections, the PRNT was applied to test serum samples collected at 1 week after disease onset from two flocks in which TMUV infection had been confirmed and a flock in which TMUV infection had been excluded. High levels of ND_50_ titer were generated from the serum samples of Shaoxing ducks in Hubei province and of Cherry Valley Pekin ducks in Shandong province, ranging from 7,943 to 79,433 and from 10,000 to 125,893 respectively. In contrast, the ND_50_ values produced from the serum samples of Pekin ducks in Inner Mongolia autonomous region ranged from 25 to 200, significantly lower than those obtained in Hubei and Shandong provinces (*P* < 0.001) (Figure 3).

**Figure 4:**
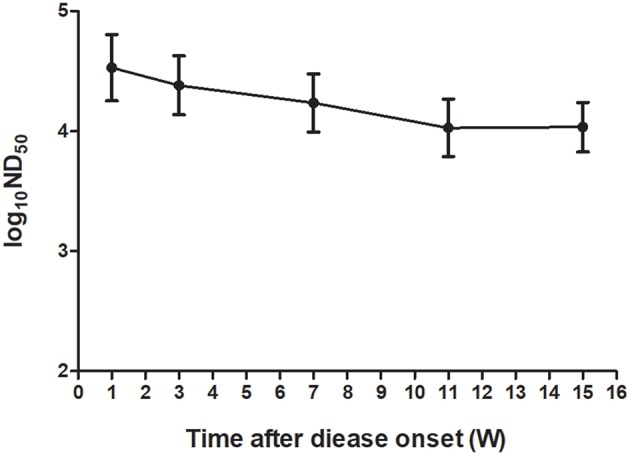
Kinetics of TMUV neutralizing antibodies in sera from a flock of Jinding ducks after an outbreak of the TMUV-related disease.

To investigate the kinetics of neutralizing antibodies in sera of ducks after TMUV natural infections, the PRNT was performed on serum samples collected at different time points from a flock of Jinding ducks in which TMUV infection had been confirmed. High neutralizing antibody titers ranging from 12,589 to 100,000 appeared in sera at 1 week after disease onset. Although the ND_50_ titers showed tendency to decline since then, high ND_50_ values ranging from 5,012 to 63,095 were detected in sera collected at 15 weeks after disease onset (Figure 4).

### Immunization With TMUV Attenuated Vaccine Elicits High-Level, Long-Term Neutralizing Antibody Response in Ducks

**Figure 5:**
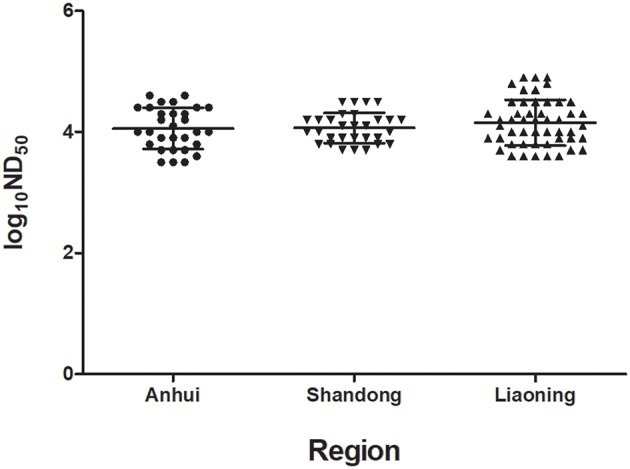
Neutralizing antibody titers in sera of ducks at 1 week after inoculation with a live-attenuated vaccine. The serum samples were collected from three flocks of ducks in different regions.

To evaluate the humoral immune response induced by TMUV attenuated vaccine, the PRNT assay was applied to test serum samples collected from three different flocks at 1 week after immunization. The ND_50_ values were generally high, ranging from 3,612 to 37,811 in sera of Cherry Valley Pekin ducks in Anhui province, from 5,012 to 31,623 in sera of Cherry Valley Pekin ducks in Shandong province, and from 3,981 to 79,432 in sera of Jinding ducks in Liaoning province (Figure 5).

**Figure 6:**
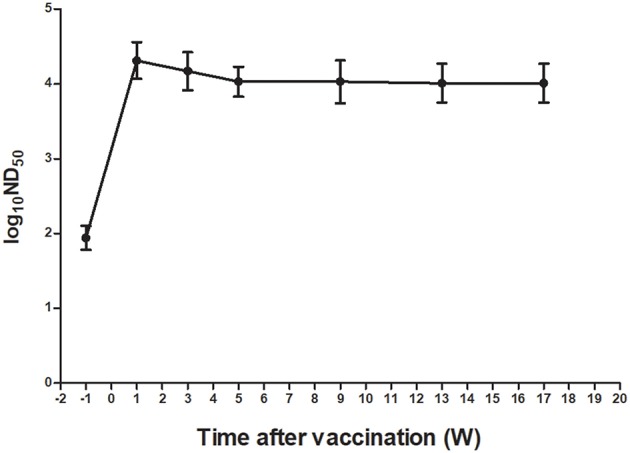
Kinetics of TMUV neutralizing antibodies in sera from a flock of Cherry Valley Pekin ducks vaccinated with a live-attenuated vaccine.

To investigate further the kinetics of neutralizing antibodies induced by TMUV attenuated vaccine, the PRNT was employed to test serum samples from a flock of Cherry Valley Pekin ducks at 1 week before immunization and at different time points after immunization. ND_50_ values detected in sera of ducks before immunization were generally lower, ranging from 50 to 158. Following vaccination, the neutralizing antibodies increased rapidly and reached 7,943–50,119 at 1 week after vaccination. On subsequent 16 weeks the ND_50_ titers declined gradually. Nevertheless, higher ND_50_ values ranging from 3,981 to 25,119 were detected at 17 weeks after vaccination (Figure 6).

## Discussion

The present study describes the development of a PRNT for the detection of neutralizing antibodies against TMUV by using a seventh cell-derived working virus. The PRNT was optimized by comparative testing of different conditions and validated by determination of intra- and inter-assay variations. The detection limit was determined to be about 1:100 to 1:1,000 of serum dilution, suggesting that the sensitivity of the test is acceptable. The specificity of the test was proven by detection of neutralizing antibodies induced by experimental and natural infections and vaccination with two TMUV attenuated strains. We were unable to carry out the differential detection of antibodies against BAGV, NTAV, and ZIKV which have been classified within the same antigenic complex group with TMUV (8, 43) since the reference antisera or strains for these flaviviruses are unavailable. However, we reasoned that the test might not exhibit cross-reactions with other flaviviruses based on the following points. First, it has been shown previously that PRNT is the most virus-specific serological test (2, 8, 11–18). Second, there have been no reports describing infections in ducks caused by other flaviviruses (e.g., BAGV, NTAV, and ZIKV) so far. We showed that the ND_50_ values obtained from negative sera can be easily distinguished from those generated from positive sera. Using TMUV antibody negative serum samples prepared in laboratory and collected from flocks uninfected by TMUV in the field, the ND_50_ values were determined to be <200, which can be regarded as a cut-off ND_50_ value. The availability of a TMUV PRNT provides a useful diagnostic tool with which investigations into TMUV antibody-mediated protection can be undertaken.

Based on clinical features and molecular detection of tissue samples collected from affected ducks, TMUV infections were confirmed to be responsible for the egg drop disease occurred in Shaoxing ducks in Hubei province, Cherry Valley Pekin ducks in Shandong province, and Jinding ducks in Liaoning province, but not for the disease occurred in Pekin ducks in Inner Mongolia autonomous region. Using the serum samples from diseased ducks, we focused on understanding the duck neutralizing antibody response to TMUV infection. We showed that high levels of neutralizing antibody (7,943–125,893) were detected in sera of ducks in Hubei and Shandong provinces, indicating that ducks exposed to TMUV infections develop strong neutralizing antibody response as early as 1 week after disease onset. The detection of low ND_50_ values in sera collected from Inner Mongolia autonomous region, indicating that these samples could be considered negative for TMUV antibodies. Thus, application of PRNT in serum samples is of help to the definitive diagnosis of the TMUV-related disease. Further detection of the kinetics of TMUV antibodies in a flock of Jinding ducks demonstrated that higher levels of TMUV neutralizing antibody induced by natural infection can persist in sera of ducks for at least 15 weeks. These findings indicate that natural infection can elicit high-level, long-lasting immunity to TMUV.

To date, a TMUV attenuated vaccine (32) has been widely used in ducks in China. In this study, therefore, we also focused on understanding humoral immunity elicited by the vaccine, which is directly relevant to control of the TMUV-related disease. We showed that neutralizing antibodies with ND_50_ titers higher than 1,258 offer adequate protection against infection of virulent TMUV. The detection of ND_50_ titers ranging from 3,612 to 79,432 in sera collected at 1 week after vaccination from three different flocks located in Anhui, Shandong, and Liaoning provinces supported the view that the licensed TMUV attenuated vaccine elicits high levels of neutralizing antibody rapidly. Further detection of the kinetics of neutralizing antibodies in sera of an immunized flock of Cherry Valley Pekin ducks in Shandong province further confirmed that TMUV attenuated vaccine leads to a rapid seroresponse from seronegative before immunization to seropositive status after vaccination and a rapid rise of TMUV-specific serum neutralizing antibody titers. Moreover, higher levels of neutralizing antibody persist for at least 17 weeks. Together these findings indicate that vaccination with the licensed live-attenuated vaccine can confer high-level, long-lasting immunity to TMUV.

Compared with those of the licensed live-attenuated vaccine (32), the PS180 strain elicited noticeably lower levels of neutralizing antibody, which might be attributed to more passages of PS180. This investigation may be of help for further studies on molecular mechanism for TMUV attenuation and antibody-mediated protection.

Taken together, we have developed a PRNT for the detection of TMUV-specific neutralizing antibodies in sera of ducks. From the investigations of TMUV antibody-positive and negative sera we conclude that the test described here is a useful diagnostic tool to serological diagnosis of TMUV infection and measurement of neutralizing antibody-mediated immunity to TMUV. Application of the test in sera from infected and immunized ducks provided evidence that neutralizing antibodies is a critical component of immunity to TMUV following both infection and vaccination, which contributes to the understanding of neutralizing antibody response to TMUV. The data obtained from detection of the kinetics of neutralizing antibodies in naturally infected and vaccinated flocks provides a scientific basis for rational use of TMUV live-attenuated vaccine.

## Data Availability Statement

All datasets generated for this study are included in the article/Supplementary Material.

## Ethics Statement

The animal study was reviewed and approved by the Animal Welfare and Ethics Censor Committee of China Agricultural University (license number CAU20171011-2). Written informed consent was obtained from the owners for the participation of their animals in this study.

## Author Contributions

DZ and JL designed the study. JL performed the experiments. LY and SQ participated in the data analysis. RM, QL, and HL participated in the animal experiments. JL, XW, and DZ wrote the manuscript.

### Conflict of Interest

The authors declare that the research was conducted in the absence of any commercial or financial relationships that could be construed as a potential conflict of interest.
