# Supplementary material for: Detection of Neutralizing Antibodies to Tembusu Virus: Implications for Infection and Immunity
Source: Front Vet Sci. 2019 Dec 10;6:442. doi: 10.3389/fvets.2019.00442 (PMC6914806; doi:10.3389/fvets.2019.00442)
Supplement: Supplementary file 1 [file Data_Sheet_1.DOCX]

| **Supplementary TABLE S1 \|** Determination of the optimal dilution of working virus | | | | | | | | | | | |
| --- | --- | --- | --- | --- | --- | --- | --- | --- | --- | --- | --- |
| Sample No.^†^ | ND_50_ titer determined with PS7 at a dilution as indicated | | | | | | | | | | |
|  | 1:7×10^3^ | | |  | 1:10^4^ | | |  | 1:2×10^4^ | | |
|  | Test 1 | Test 2 | Test 3 |  | Test 1 | Test 2 | Test 3 |  | Test 1 | Test 2 | Test 3 |
| 1 | 13 | 20 | 20 |  | 25 | 20 | 25 |  | 13 | 80^‡^ | 12 |
| 2 | 40 | 40 | 32 |  | 50 | 32 | 63 |  | 32 | 63 | 40 |
| 3 | 20 | 13 | 25 |  | 32 | 16 | 13 |  | 13 | 12 | 63^‡^ |
| 4 | 40 | 32 | 25 |  | 40 | 32 | 32 |  | 40 | 25 | 40 |
| 5 | 79431 | 125893 | 63096 |  | 63096 | 50112 | 50112 |  | 50112 | 100000 | 63096 |
| 6 | 10000 | 12589 | 7943 |  | 31622 | 25119 | 39811 |  | 12589 | 79433^‡^ | 10000 |
| 7 | 31623 | 39811 | 25119 |  | 12589 | 25119 | 12589 |  | 50119^‡^ | 10000 | 12589 |
| 8 | 12589 | 10000 | 10000 |  | 7943 | 12589 | 15848 |  | 10000 | 7943 | 12589 |
| 9 | 25119 | 31622 | 31622 |  | 19953 | 25119 | 31622 |  | 79433^‡^ | 19953 | 25119 |
| 10 | 63096 | 79433 | 63096 |  | 79433 | 50119 | 50119 |  | 63096 | 12589^‡^ | 79433 |
| ^†^Samples 1–4 indicate negative sera collected from 1-day-old ducklings. Samples 5–10 indicate antisera against TMUV Y4.  ^‡^Outside ± 3-fold of the median titer of three results obtained in triplicates. | | | | | | | | | | | |

| **Supplementary TABLE S2 \|** Determination of the optimal virus-serum neutralization time | | | | | | | | | | | | | | | | | | | |
| --- | --- | --- | --- | --- | --- | --- | --- | --- | --- | --- | --- | --- | --- | --- | --- | --- | --- | --- | --- |
| Sample No.^†^ | ND_50_ titer determined with neutralization time as indicated | | | | | | | | | | | | | | | | | | |
|  | 30 min | | |  | 45 min | | |  | 60 min | | |  | 75 min | | |  | 90 min | | |
|  | Test 1 | Test 2 | Test 3 |  | Test 1 | Test 2 | Test 3 |  | Test 1 | Test 2 | Test 3 |  | Test 1 | Test 2 | Test 3 |  | Test 1 | Test 2 | Test 3 |
| 1 | 10 | 16 | 10 |  | 13 | 20 | 13 |  | 25 | 20 | 25 |  | 13 | 20 | 20 |  | 20^‡^ | 100 | 126 |
| 2 | 13 | 40^‡^ | 16 |  | 40 | 40 | 32 |  | 50 | 32 | 63 |  | 32 | 63 | 40 |  | 80 | 80 | 80 |
| 3 | 16 | 25 | 25 |  | 32 | 40 | 50 |  | 32 | 20 | 32 |  | 40 | 25 | 63^‡^ |  | 100 | 32^‡^ | 100 |
| 4 | 13 | 10 | 50^‡^ |  | 40 | 50 | 50 |  | 40 | 32 | 40 |  | 40 | 25 | 40 |  | 63 | 50 | 100 |
| 5 | 12589 | 7943 | 50119^‡^ |  | 79431 | 125893 | 63096 |  | 63096 | 50119 | 50119 |  | 50112 | 50112 | 63096 |  | 63096 | 79433 | 19958^‡^ |
| 6 | 12589 | 19953 | 25119 |  | 25112 | 19953 | 19953 |  | 31622 | 25119 | 39811 |  | 15849 | 31622 | 31622 |  | 79433 | 79433 | 25119^‡^ |
| 7 | 31623^‡^ | 10000 | 7943 |  | 25119 | 39811 | 25119 |  | 12589 | 25119 | 15848 |  | 15849 | 10000 | 15849 |  | 50119 | 50119 | 25119 |
| 8 | 7943 | 7943 | 10000 |  | 12589 | 12589 | 7943 |  | 10000 | 7943 | 7943 |  | 10000 | 7943 | 10000 |  | 31622 | 31622 | 19953 |
| 9 | 25119^‡^ | 5012 | 7943 |  | 19953 | 25119 | 31622 |  | 19953 | 19953 | 25119 |  | 10000 | 10000 | 12589 |  | 50119 | 79433 | 79433 |
| 10 | 50112^‡^ | 12589 | 12589 |  | 63096 | 63096 | 50112 |  | 79433 | 63096 | 79433 |  | 63096 | 63096 | 79433 |  | 100000 | 31622^‡^ | 125893 |
| ^†^Samples 1–4 indicate negative sera collected from 1-day-old ducklings. Samples 5–10 indicate antisera against TMUV Y4.  ^‡^ Outside ± 3-fold of the median titer of three results obtained in triplicates. | | | | | | | | | | | | | | | | | | | |

| **Supplementary TABLE S3 \|** Determination of the optimal incubation time for adsorption of virus-serum inoculum to the cell surface | | | | | | | | | | | | | | | |
| --- | --- | --- | --- | --- | --- | --- | --- | --- | --- | --- | --- | --- | --- | --- | --- |
| Sample No.^†^ | ND_50_ titer determined with adsorption time as indicated | | | | | | | | | | | | | | |
|  | 30 min | | |  | 40 min | | |  | 50 min | | |  | 60 min | | |
|  | Test 1 | Test 2 | Test 3 |  | Test 1 | Test 2 | Test 3 |  | Test 1 | Test 2 | Test 3 |  | Test 1 | Test 2 | Test 3 |
| 1 | 25 | 16 | 25 |  | 13 | 20 | 13 |  | 25 | 20 | 25 |  | 13 | 16 | 20 |
| 2 | 32 | 40 | 32 |  | 40 | 40 | 32 |  | 50 | 32 | 63 |  | 32 | 63 | 40 |
| 3 | 100 | 25^‡^ | 80 |  | 20 | 13 | 25 |  | 32 | 16 | 13 |  | 13 | 32 | 32 |
| 4 | 25 | 20 | 50 |  | 40 | 50 | 63 |  | 40 | 32 | 32 |  | 40 | 25 | 40 |
| 5 | 12589 | 15849 | 50119^‡^ |  | 79431 | 125893 | 63096 |  | 63096 | 50112 | 50112 |  | 50119 | 100000 | 63096 |
| 6 | 79433 | 63096 | 100000 |  | 10000 | 12589 | 12589 |  | 31622 | 25119 | 39811 |  | 12589 | 25119 | 25119 |
| 7 | 50119^‡^ | 10000 | 12589 |  | 10000 | 10000 | 12589 |  | 12589 | 25119 | 12589 |  | 15848 | 10000 | 15848 |
| 8 | 31622 | 31622 | 19953 |  | 7943 | 10000 | 15849 |  | 7943 | 7943 | 10000 |  | 10000 | 10000 | 7943 |
| 9 | 79433 | 19953^‡^ | 79433 |  | 25119 | 25119 | 25119 |  | 19953 | 25119 | 19953 |  | 25119 | 19953 | 19953 |
| 10 | 50119 | 50119 | 12589^‡^ |  | 63096 | 50119 | 79433 |  | 63096 | 79433 | 100000 |  | 79433 | 63096 | 59119 |
| ^†^Samples 1–4 indicate negative sera collected from 1-day-old healthy ducklings. Samples 5–10 indicate antisera against TMUV Y4.  ^‡^ Outside ± 3-fold of the median titer of three results obtained in triplicates. | | | | | | | | | | | | | | | |

**84 h**

**78 h**

**72 h**

**66 h**

**60 h**


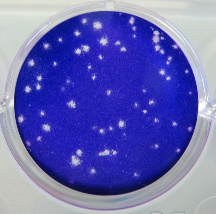

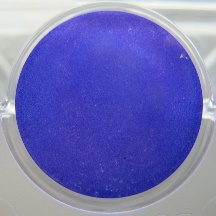

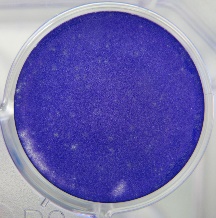

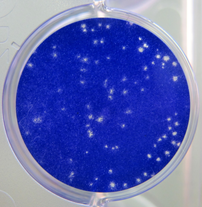

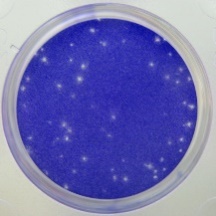


**Supplementary Figure S1**. Determination of the optimal incubation time after the plates received overlay medium.
